# Screening for hypertension in the inpatient environment (SHINE): a prospective diagnostic accuracy study among adult hospital patients

**DOI:** 10.1136/bmjopen-2025-107038

**Published:** 2026-01-19

**Authors:** Laura Catherine Armitage, Cristian Roman, Beth K Lawson, Adam Mahdi, Christopher Biggs, Louise Young, Holly Edmundson, Thomas Fanshawe, Lionel Tarassenko, Andrew Farmer, Peter J Watkinson

**Affiliations:** 1 Nuffield Department of Primary Care Health Sciences, University of Oxford, Oxford, UK; 2 Institute of Biomedical Engineering, University of Oxford, Oxford, UK; 3 Department of Engineering, University of Oxford, Oxford, UK; 4 Oxford Internet Institute, University of Oxford, Oxford, UK; 5 Nuffield Department of Clinical Neurosciences, University of Oxford, Oxford, UK; 6 Gastroenterology Clinical Trials Facility, Oxford University Hospitals NHS Foundation Trust, Oxford, UK; 7 Engineering Science, University of Oxford, Oxford, UK

**Keywords:** Hypertension, Blood Pressure, PREVENTIVE MEDICINE, PUBLIC HEALTH

## Abstract

**Background:**

Hypertension is the leading risk factor for death globally. Undiagnosed hypertension is common, but the incidence in hospitalised patients is unclear. There are calls for universal facility-based screening for hypertension among all attending patients. The hospital inpatient setting, where blood pressure (BP) is measured routinely and repeatedly, presents an ideal opportunity. However, international hypertension guidelines do not include inpatient BP thresholds for diagnostic or treatment purposes. We investigated the performance of current UK community BP thresholds for diagnosing hypertension in the hospital setting.

**Objectives:**

Investigate the diagnostic performance of the current UK ambulatory BP diagnostic thresholds for systolic and diastolic hypertension in the hospital setting against the reference test of community-based ambulatory BP monitoring (ABPM).

**Design:**

A prospective diagnostic accuracy study.

**Setting:**

Hospital inpatients admitted to three UK centres were approached. Follow-up ABPM was delivered in the community.

**Participants:**

Eligible patients were aged between 18 and 80 years, with no prior diagnosis of, or prescription for hypertension, and whose mean cumulative daytime BP was 120 mm Hg to 179 mm Hg systolic and ≤109 mm Hg diastolic from the 24th hour of their hospital admission.

**Interventions:**

Participants received 24-hour ABPM 4–26 weeks post-discharge, as the reference test for hypertension, with UK diagnostic thresholds of an average daytime BP of ≥135 mm Hg systolic and ≥85 mm Hg diastolic applied. Participants found to be severely hypertensive at the ABPM fitting appointment were also considered reference-test positive but did not proceed with ABPM.

**Primary and secondary outcome measures:**

The diagnostic performance of a mean daytime in-hospital BP of ≥135 mm Hg systolic or ≥85 mm Hg diastolic (index test) for the prediction of hypertension diagnosed on ABPM (reference test) was assessed using sensitivity, specificity, positive predictive value (PPV) and negative predictive value (NPV) as primary outcome measures. Additionally, we explored the accuracy of a range of alternative in-hospital systolic and diastolic BP thresholds against the same reference test.

**Results:**

351 participants were enrolled and 206 completed the study protocol. The average age of the 206 participants was 53 years, 55% were male, and 91 (44%) had daytime community hypertension on ABPM reference testing. Of 107 participants with raised in-hospital daytime BP, 59 (55%) had daytime community hypertension. When assessing the performance of the index test for detecting daytime community hypertension, sensitivity was 65% (59/91, 54% to 75%) and specificity was 58% (67/115, 49% to 67%). The PPV was 55% (59/107, 45% to 65%) and NPV was 68% (67/99, 58% to 77%), respectively. A further 45/206 participants (23%) had night-time community hypertension when assessed using European diagnostic thresholds for nocturnal hypertension (120 mm Hg systolic or 70 mm Hg diastolic), while 25/107 of those with raised in-hospital daytime BP (23%) had night-time community hypertension. When assessing the performance of the index test for detecting either day or night-time community hypertension, sensitivity was 62% (84/135, 53% to 70%) and specificity was 68% (48/71, 55% to 78%). The PPV was 79% (84/107, 70% to 86%) and NPV was 48% (48/99, 38% to 59%).

**Conclusions:**

Undiagnosed hypertension is common in hospitalised patients, particularly those with raised in-hospital BP. While in-hospital BP alone is an imperfect predictor and should not be used as a stand-alone diagnostic test, this could serve as a trigger for further assessment of BP in the community after discharge.

**Trial registration number:**

The study protocol was registered with the ISCTRN Registry (ISRCTN80586284).

**STRENGTHS AND LIMITATIONS OF THIS STUDY**

- This is a prospective diagnostic accuracy study, performed and reported according to Standards for Reporting Diagnostic Accuracy.
- Recruitment was from 3 UK centres in the South of England.
- Patient sampling was stratified to achieve a cohort with a range of index test results.
- The application of the index test, its conduct and the population it was tested in are as close as possible to how the test would be applied in a real-world setting with the target population and without knowledge of the reference standard test result.
- The reference standard test is likely to correctly identify the target condition, as we used the long-established and gold-standard method of hypertension diagnosis in the form of ambulatory blood pressure monitoring over a 24-hour period.

## Introduction

 Across the globe, hypertension is the leading risk factor for death, accounting for 12.8% of annual mortality.^1^ There have been recent calls for ‘universal facility-based’ hypertension screening, moving efforts to identify undiagnosed hypertension away from mass screening programmes in the community to healthcare facilities.^2^ A marked proportion of hospital inpatients have undiagnosed hypertension,^3^,^5^ yet follow-up of patients with raised blood pressure (BP) in hospital is poor.^6^,^8^ This presents a missed opportunity for facility-based screening to identify the approximately 7.5 million people currently living with untreated hypertension in England alone.^9^

Clinicians may not be recommending follow-up BP assessment due to a belief that raised in-hospital BP is caused by pain,^10^ anxiety^11^ or white coat syndrome.^12^ While this may be true for a proportion of patients, a recent meta-analysis of primary studies demonstrated that almost half of those with a raised BP in the Emergency Department setting have sustained hypertension when followed up in the community after discharge.^13^ However, none of the studies included in this review were diagnostic accuracy studies, or collected data such that measures of diagnostic accuracy could be calculated or appropriate diagnostic thresholds discerned, as they only recruited and reported on participants whose BP exceeded a single threshold for systolic and diastolic BP. Additionally, none included BP data from the inpatient setting, where the majority of patients’ BP data are collected.

This gap in evidence underpins the fact that the UK, European and American guidelines do not include any guidance for clinicians in assessing for the presence of hypertension in the hospital setting unless it is severe, and thresholds for diagnosis are suggested only for the outpatient clinic or home settings.^14^,^16^ It may be that these thresholds for the community and outpatient settings could be applied in the hospital setting, but it is not known whether measurements of individuals’ in-hospital BP accurately reflect their BP in the community.

Until recently, in-hospital BP measurements were hand-recorded on paper, making screening patients with elevated BP for either research or clinical purposes unmanageable. Many hospitals now use electronic systems for recording vital signs such as BP and pulse.^17^ This has paved the way for automated identification of patients whose BP in hospital exceeds a defined threshold. This, in turn, could help patients become diagnosed with, and start treatment for, hypertension sooner.

### Study objectives and hypothesis

The objective of this study was to investigate the diagnostic performance of the current UK ambulatory BP diagnostic thresholds for systolic and diastolic hypertension in the hospital setting against the reference test of community-based ambulatory BP monitoring (ABPM).^14^ Additionally, we explored the accuracy of a range of alternative in-hospital BP systolic and diastolic BP thresholds against the same reference test.

## Methods and analysis

A prospective, diagnostic accuracy study, conducted in three UK NHS hospital trusts. The study is reported to the Standards for Reporting Diagnostic Accuracy.^18^ The study protocol was both registered with the ISCTRN Registry (ISRCTN80586284) and published.^19^

Ethical approval was provided by the National Health Service Health Research Authority South Central-Oxford B Research Ethics Committee (19/SC/0026).

### Study setting

Participants were recruited from Oxford University Hospitals NHS Foundation Trust, UK between August 2019 and November 2022, South Warwickshire University NHS Foundation Trust, UK between July 2021 and January 2022 and Royal Berkshire NHS Foundation Trust, UK between February 2022 and August 2022. Study follow-up for reference test ABPM occurred between November 2019 and May 2023 via one of a: (1) Face-to-face appointment at a primary care centre, (2) Telemedicine appointment or (3) Visit to the participant’s home.

### Study population

Eligible patients were aged between 18 and 80 years, admitted to one of the three trusts, with no prior diagnosis of, or prescription for hypertension, and whose mean cumulative daytime BP was 120 mm Hg to 179 mm Hg systolic and ≤109 mm Hg diastolic from the 24th hour of their hospital admission. Individuals were required to have a minimum of three BP measurements taken over a minimum period of 24 hours, with at least one BP being recorded during night-time hours (22:00 to 06:59) and at least two being recorded during day-time hours (07:00 to 21:59).^19^ The requirement for a nocturnal BP measurement was defined to enable future work analysing the diagnostic accuracy of nocturnal measurements in hospital. BP measurements taken in intensive or high-dependency care, operating theatres and maternity units were not included, and patients could not be deemed eligible while in any of these care settings. Additional exclusion criteria were: current, intended or recent pregnancy; previous hypertension diagnosis and/or prescribed anti-hypertensive medication; cause for index admission being associated with end-organ damage related to severe hypertension (including but not limited to heart failure, myocardial infarction, stroke, hypertensive encephalopathy); and estimated glomerular infiltration rate <30 mL/min. Full inclusion and exclusion criteria are provided in the published study protocol.^19^

### Screening procedure

We designed and automated an algorithm to screen for eligible patients that was run against electronic patient records of the included hospital trusts every 24 hours. The algorithm screened based on electronic records of BP measurements, recorded diagnostic codes and prescribed medications. The BP measurements were entered manually as part of usual care, into the hospital’s electronic system for vital-sign recording. All in-hospital BP monitoring equipment used in the study was purchased, maintained and calibrated in line with each hospital’s normal medical devices management policy. The patient notes of potentially eligible patients flagged by the algorithm were then further manually screened by the clinical research team. If no further exclusion criteria were found, the patients were approached.

Patients gave informed, written consent prior to study participation.

### Participant sampling

We stratified patients into one of 5 consecutive systolic BP profiles (120–129 mm Hg, 130–139 mm Hg, 140–149 mm Hg, 150–159 mm Hg, 160–179 mm Hg), based on average day-time in-hospital BP at the point of the screening algorithm identifying them as potentially eligible.

Patients were screened by the clinical research team in a consecutive manner, but those with the higher eligible mean day-time BPs were prioritised to be sampled for recruitment as a retrospective analysis of in-hospital BP (unpublished data in preparation for the study) indicated higher BP profiles had the lowest prevalence among the eligible population and were therefore likely to be the most challenging to recruit.

### Data collection

Qualified clinical researchers collected and managed study data using custom-made baseline case report forms in REDCap (Research Electronic Data Capture, https://projectredcap.org/software/) hosted at the University of Oxford,^20 21^ using a combination of medical record review and patient self-report. For example, information on ethnicity, family history and previous prescribed medications for hypertension was collected via patient self-report, while BP, age, sex, ICD-10 codes and current prescription information were obtained from the patient health records.

### Diagnostic test methods

#### Index diagnostic test

The index test was cumulative mean in-hospital daytime BP from the point of admission to an eligible ward until the end of at least the first 24-hour period in which the screening algorithm identified a patient as being eligible for approach, re-estimated up to day 10 of the admission, or discharge (whichever occurred first). The BP measurements were performed by the direct clinical care team as part of usual care during the participants’ hospital admissions, with no element (eg, frequency of measurements, participant position) being controlled or observed by the study team. In the primary analysis, index test results for systolic and/or diastolic hypertension were considered to be positive (raised mean in-hospital daytime BP) if ≥135 mm Hg systolic and/or ≥85 mm Hg diastolic, respectively, based on current UK National Institute for Health and Care Excellence (NICE) diagnostic thresholds for ABPM.^14^

Additionally, we explored the accuracy of a range of alternative in-hospital BP systolic and diastolic BP thresholds (ranging from 125 to 160 mm Hg for systolic BP and 70–90 mm Hg for diastolic BP) against the same reference test, to assess whether there may be alternative systolic and diastolic BP thresholds for the in-hospital settings with greater predictive performance.

#### Reference standard diagnostic test

The reference diagnostic test for hypertension was a daytime ambulatory BP ≥135 mm Hg systolic or ≥85 mm Hg diastolic, based on the current NICE diagnostic thresholds for ABPM.^14^ Reference testing was performed without knowledge of the index test result. Patients found to have severe hypertension at their ABPM fitting appointment (systolic BP ≥180 mm Hg and/or diastolic BP ≥110 mm Hg)^14^ were considered reference-test positive and to have ‘completed’ ABPM reference test follow-up (but did not proceed with ABPM and were referred to their primary care provider urgently for further assessment). We also assessed the proportion of patients with isolated elevated nocturnal BP (mean night-time systolic BP ≥120 mm Hg and/or mean night-time diastolic BP ≥70 mm Hg) as defined by the European diagnostic guidelines for hypertension.^15^ We defined the severity of hypertension on reference diagnostic testing as follows: (1) Stage 1 hypertension: average systolic BP ≥135 and ≤149 mm Hg or average diastolic BP ≥85 and ≤94 mm Hg diastolic, (2) Stage 2 hypertension: average systolic BP ≥150 and ≤159 mm Hg or average diastolic BP ≥95 and ≥104 mm Hg and (3) Severe hypertension: average systolic BP ≥160 mm Hg or average diastolic BP ≥105 on ABPM, or systolic BP ≥180 mm Hg or diastolic BP ≥110 mm Hg at ABPM fitting appointment. Elevated night-time BP was defined as an average night-time systolic BP ≥120 mm Hg or ≥70 mm Hg diastolic.

Reference standard results were not available to performers of the index test. Follow-up ABPM was scheduled with each participant for approximately 8 weeks (permitted from 4 to 26 weeks) following discharge from hospital and performed using a validated automated Mobil-O-Graph ABPM (IEM GmbH, Stolberg, Germany), calibrated to manufacturer standards. Fitting of the ABPM was performed either face-to-face or remotely, by a qualified research clinician, appropriately trained in the National Institute for Health’s Good Clinical Practice and to perform ABPM. The research clinicians performing ABPM were blinded to the result of the index test. The remote fitting and removal processes were designed and added as part of an amended protocol (ethical approval granted) to reduce infection risk to participants and researchers during the COVID-19 pandemic. The processes of remote fitting and removal have been described in detail elsewhere.^22^ In brief, where remote fitting of the ABPM was performed, participants also received remote screening for pulse irregularity using a KardiaMobile ECG device and associated KardiaMobile app (AliveCor, Mountain View, California).

The ABPM was programmed to measure BP twice per hour during participants’ self-reported anticipated waking hours and once per hour during sleeping hours, for a minimum period of 24 hours.^23^ A minimum of 70% of the daytime and 70% of the night-time ABPM recordings were required to be successful in order to calculate the mean BP for daytime and night-time.^15^ Because the diagnostic assessment for hypertension in this study was based on the UK NICE Guidelines which assess daytime readings only, when fewer than 70% of the daytime recordings were successful, the results were considered invalid and the participant was asked to wear the monitor for a further 24-hour period. We stipulated a priori that participants needed a minimum of 14 successful waking-time ABPM measurements for the mean day-time BP to be calculated, as per the British and Irish Hypertension Society Standard Operating Procedure for ABPM.^23^ In the instance of two 24-hour episodes of ABPM not collecting sufficient day-time BP data, the participant and their registered general practitioner were informed and the participants excluded from the analysis.

#### Sample size

The target sample size was calculated using estimates of expected prevalence of hypertension at follow-up, owing to the novel design of this study. A sample size of 200 participants with complete follow-up was therefore derived to permit detection of a 20% rate of hypertension at follow-up, with a 95% CI width of approximately ±5.52%. Throughout the study, we monitored drop-out rates such that recruitment only ceased once the study team were confident 200 follow-up ABPMs would be achieved.

### Analysis

All analyses were performed using R Statistical Software (V.4.1.2; R Core Team 2021). A complete case analysis was performed by excluding patients without an observed test result. The nature of the diagnostic tests and our inclusion criteria meant participants were not expected to have an indeterminate index or reference standard test result. We assessed the diagnostic performance of the predefined in-hospital BP index test thresholds of ≥135 mm Hg systolic and ≥85 mm Hg diastolic (‘raised mean in-hospital blood pressure’) by estimating the sensitivity, specificity, positive predictive values (PPVs) and negative predictive values (NPVs) relative to hypertension determined by mean daytime ABPM. We constructed Receiver Operator Characteristic (ROC) curves within R Studio^24^ using daytime in-hospital mean systolic and diastolic BP (index test) as continuous measurements to predict the diagnosis of systolic and diastolic hypertension respectively, to assess variability in diagnostic accuracy as in-hospital BP diagnostic thresholds (index test) were varied. The Area Under the Curve for the Receiver Operator Characteristic (AUROC) was calculated, with a 95% CI, to estimate the discriminatory power of the diagnostic tests.^25^

### Patient and public involvement

Patients were first involved in this research at the design stage. Patient suggestions and opinions on screening electronic health records for study eligibility and screening routinely collected data for undiagnosed disease were gathered. It became clear that among all the patient groups who contributed, there was a strong expectation that routinely collected data is used to screen for disease, and that patients would actively want to be included in research as they felt there was potential for tangible benefit to the patients involved, through being better informed of their health and BP. They felt the burden of participation would be acceptable. Patient representatives informed us they would like to see screening tests like this be introduced and be inclusive as far as possible but also tailored to need, so that those patients most likely to benefit are specifically targeted by a screening intervention. When we were required to pivot the delivery of the study follow-up to being remote during the COVID-19 pandemic, this process was thoroughly assessed, step-by-step by key stakeholders, including a patient representative from the British Heart Foundation’s Heart Voices group, through a formal Failure Modes and Effects Analysis. This process has been published elsewhere.^22^

## Results

**Figure 1:**
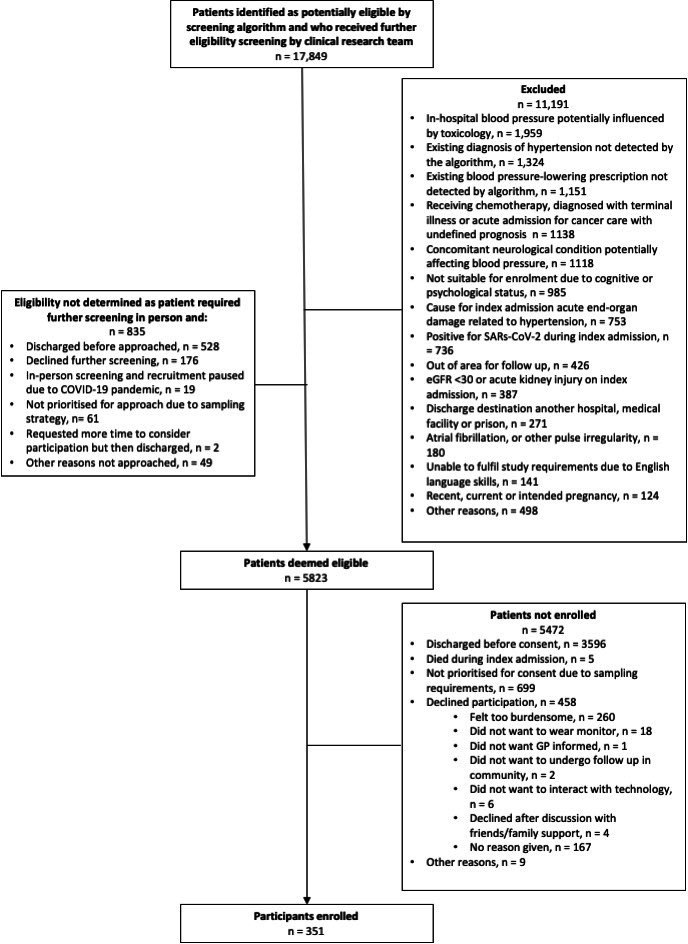
Participant flow diagram. GP, general practitioner.

During the recruitment periods for the three sites, 17 849 patients were identified via the automated screening algorithm as being potentially eligible and underwent further screening by the clinical research teams. Three hundred and fifty-one participants were recruited to the study. Reasons for exclusion are shown (figure 1). From the enrolled 351 study participants, 145 participants did not proceed to ABPM reference testing; of these, 73 participants chose to withdraw from the study, while 72 were lost to follow-up (with 16 being unable to complete the study protocol due to suspension of studies during the COVID-19 pandemic). Reasons for withdrawal are further shown in figure 1. Height data were missing for nine participants where their clinical condition did not allow measurement. The R script for all analyses and results reported is provided in online supplemental information file 2.

A total of 200 participants completed 24-hour ABPM reference testing. A further six had severe hypertension at their ABPM fitting appointment and so were considered reference test positive.

Baseline characteristics for recruited participants are presented in table 1, separately for those who underwent reference testing and those who withdrew from the study or were lost to follow-up. Average age, body mass index, in-hospital systolic and diastolic BP were similar between those who completed the reference diagnostic test follow-up and those who did not, as were the proportion that were male and the proportion of those with diabetes mellitus.

**Table 1:** Baseline characteristics of the recruited cohort

Baseline characteristics		Cohort with reference diagnostic test (206)	Withdrew from study or lost to follow-up (145)
Age	Median (IQR)	53 (43 to 64)	53 (42 to 62)*
	Mean (SD)	52.96 (14.64)	51.67 (14.49)*
Sex, % male (n)		54.9 (113)	55.5 (76)*
Ethnicity, % (n)		White 96% (198) Indian 1% (3) Chinese 1% (2) Other Asian background 1% (2) Other ethnic group 0.5% (1)	White 91% (127) White and black African or white and black Caribbean 4% (5) Indian 3% (4) Black, African or Caribbean 3% (4) (n missing=5)
Body mass index (mean, SD)		28.65 (5.98)†	28.63 (6.74)†
History of diabetes mellitus, % (n)		4.3 (9)	5.1 (7)*
Average in-hospital systolic BP, mm Hg, mean (SD)		136.43 (12.63)	138.02 (13.60)
Average in-hospital diastolic BP, mm Hg, mean (SD)		77.66 (9.33)	76.99 (9.41)

Baseline characteristics of recruited cohort divided into those who completed reference testing, comprising 200 people with ambulatory blood pressure monitoring (ABPM) data and six who were found to have severe hypertension at their ABPM fitting appointment.

* Denotes data missing for three enrolled individuals who withdrew or were lost to the study shortly after enrolment.

† Denotes data missing for six patients in the cohort and three who withdrew or were lost to follow-up.

The proportion of patients whose mean in-hospital day-time systolic BP fell into one of the five bands from which participants were sampled, in both the screened and enrolled cohorts, are presented in online supplemental table S1.

The median time between hospital discharge and ABPM for all participants was 56 days (IQR 41 to 67). Median time between hospital discharge and ABPM was 53 days (IQR 40 to 75) for those correctly classified (true positives and true negatives), and 61 days (IQR 47 to 91) for those incorrectly classified (false positives and false negatives).

Among the 206 participants who completed ABPM reference testing, daytime community hypertension was detected in 91 (44%) while isolated community nocturnal hypertension was detected in a further 45 (23%). For the 107 patients meeting index diagnostic test criteria (raised mean daytime in-hospital BP), daytime community hypertension was detected in 59 (55%), while isolated community nocturnal hypertension was detected in a further 25 (23%). A breakdown of the type of hypertension detected (systolic, diastolic, stage 1, stage 2 and severe) is presented in table 2. In this table, daytime figures include participants who also had elevated night-time BP, while night-time figures include only participants who did not have daytime hypertension and would be additional cases to be identified if European guidelines were applicable in the UK.

**Table 2:** Prevalence of hypertension and isolated elevated night-time blood pressure among the participants who completed reference testing

ABPM classification		Number of participants (n=206)	Number of participants meeting index diagnostic text (‘raised in-hospital blood pressure’) (n=107)
Daytime			
Systolic or diastolic daytime hypertension	Of any severity	91 (44%)	59 (55%)
	Stage 1	68 (33%)	40 (37%)
	Stage 2	16 (6%)	14 (13%)
	Severe	7 (3%)	5 (5%)
Systolic daytime hypertension		55 (27%)	40 (37%)
Diastolic daytime hypertension		68 (33%)	43 (40%)
Systolic and diastolic daytime hypertension		32 (16%)	24 (22%)
Night-time			
Elevated systolic or diastolic night-time blood pressure*		45 (23%)	25 (23%)
Elevated systolic night-time blood pressure*		22 (11%)	14 (13%)
Elevated diastolic night-time blood pressure*		40 (20%)	22 (21%)
Elevated systolic and diastolic night-time blood pressure*		17 (9%)	11 (10%)
Daytime and night-time			
Normotension		70 (34%)	23 (21%)

Prevalence of hypertension and elevated night-time blood pressure among the participants who completed reference testing (24-hour ABPM, or detected as having severe hypertension at ABPM fitting appointment), classified by type (systolic or diastolic) and severity of hypertension.

* Calculated on the number of participants who completed ABPM (n=200), the denominator excludes those participants who had hypertension at ABPM fitting, but includes those participants who had daytime hypertension.

ABPM, ambulatory blood pressure monitoring.

### Index test performance

When defining the index test for daytime community systolic or diastolic hypertension as a combined threshold of either a daytime in-hospital systolic BP of ≥135 mm Hg or diastolic in-hospital BP of ≥85 mm Hg, for detecting the presence of daytime systolic or diastolic hypertension in the community, the sensitivity was 65% (59/91, 95% CI 54% to 75%) and specificity was 58% (67/115, 95% CI 49% to 67%), respectively, while the PPV was 55% (59/107, 95% CI 45% to 65%) and NPV was 68% (67/99, 95% CI 58% to 77%), respectively (table 3). When assessing the performance of the index test for detecting daytime or night-time community hypertension, sensitivity was 62% (84/135, 53% to 70%) and specificity was 68% (48/71, 55% to 78%). The PPV was 79% (84/107, 70% to 86%) and NPV was 48% (48/99, 38% to 59%) (table 4).

**Table 3:** Contingency matrix assessing diagnostic performance of the index test for identifying daytime community hypertension

		Systolic or diastolic daytime hypertension on ABPM		Measures
		+	–	
In-hospital systolic blood pressure ≥135 mm Hg or diastolic blood pressure ≥85 mm Hg	+	59	48	PPV=55% (59/107), 95% CI 45% to 65%
	–	32	67	NPV=68% (67/99), 95% CI 58% to 77%
Measures		Sensitivity=65% (59/91), 95% CI 54% to 75%	Specificity=58% (67/115), 95% CI 49% to 67%	

The index test for daytime community systolic or diastolic hypertension is a combined threshold of either a daytime in-hospital systolic blood pressure of ≥135 mm Hg or a diastolic in-hospital blood pressure of ≥85 mm Hg, and the reference test is the presence of daytime systolic or diastolic hypertension on ambulatory blood pressure monitoring using the same thresholds.

ABPM, ambulatory blood pressure monitoring; NPV, negative predictive value; PPV, positive predictive value.

**Table 4:** Contingency matrix assessing diagnostic performance of the index test for identifying daytime or night-time community hypertension

		Systolic or diastolic daytime or night-time hypertension on ABPM		Measures
		+	–	
In-hospital systolic blood pressure ≥135 mm Hg or diastolic blood pressure ≥85 mm Hg	+	84	23	PPV=79% (84/107), 95% CI 70% to 86%
	–	51	48	NPV=48% (48/99), 95% CI 38% to 59%
Measures		Sensitivity=62% (84/135), 95% CI 53% to 70%	Specificity=68% (48/71), 95% CI 55% to 78%	

The index test for daytime or night-time community systolic or diastolic hypertension being a combined threshold of either a daytime in-hospital systolic blood pressure of ≥135 mm Hg or diastolic in-hospital blood pressure of ≥85 mm Hg, and reference test the presence being daytime or night-time systolic or diastolic hypertension on ambulatory blood pressure monitoring using the same thresholds for daytime and a threshold of ≥120 mm Hg systolic or ≥70 mm Hg diastolic for night-time.

ABPM, ambulatory blood pressure monitoring; NPV, negative predictive value; PPV, positive predictive value.

The correlations between mean in-hospital daytime systolic and diastolic BP and mean community daytime and night-time systolic and diastolic BP are shown in online supplemental figure S1A,B. Sensitivity, specificity, PPVs and NPVs for a range of alternative in-hospital daytime systolic and diastolic cut-offs for predicting systolic and diastolic daytime hypertension in the community are presented in tables5  6. ROC curves using day-time in-hospital mean systolic and diastolic BP as continuous measurements to predict the diagnosis of systolic and diastolic hypertension respectively are shown in online supplemental figures S2 and S3. The AUROC for in-hospital day-time systolic BP as the predictor for systolic hypertension was 0.69 (95% CI 0.61 to 0.78) while the AUROC for in-hospital day-time diastolic BP as the predictor for diastolic hypertension was 0.70 (95% CI 0.62 to 0.78).

**Table 5:** Diagnostic performance of in-hospital daytime systolic blood pressure as the index test for out-of-hospital daytime systolic hypertension

In-hospital Systolic BP threshold	Sensitivity (95% CI)	Specificity (95% CI)	PPV (95% CI)	NPV (95% CI)
≥120 mm Hg	1.0 (1.0 to 1.0) 54/54	0.0 (0.0 to 0.0)	0.26 (0.2 to 0.32)	N/A (N/A to N/A)
		0/152	54/206	0/0
≥125 mm Hg	0.89 (0.81 to 0.97) 48/54	0.26 (0.19 to 0.33)	0.3 (0.23 to 0.37)	0.87 (0.77 to 0.97)
		39/152	48/161	39/45
≥130 mm Hg	0.8 (0.69 to 0.9) 43/54	0.43 (0.36 to 0.51)	0.33 (0.25 to 0.41)	0.86 (0.78 to 0.94)
		66/152	43/129	66/77
≥135 mm Hg*	0.72 (0.6 to 0.84)	0.57 (0.49 to 0.65)	0.38 (0.28 to 0.47)	0.85 (0.78 to 0.92)
	39/54	87/152	39/104	87/102
≥140 mm Hg	0.59 (0.46 to 0.72) 32/54	0.69 (0.62 to 0.76)	0.41 (0.3 to 0.51)	0.83 (0.76 to 0.89)
		105/152	32/79	105/127
≥150 mm Hg	0.28 (0.16 to 0.4) 15/54	0.92 (0.88 to 0.96)	0.56 (0.37 to 0.74)	0.78 (0.72 to 0.84)
		140/152	15/27	140/179
≥160 mm Hg	0.13 (0.04 to 0.22) 7/54	0.98 (0.96 to 1.0) 149/152	0.7 (0.42 to 0.98)	0.76 (0.7 to 0.82)
			7/10	149/196

* Denotes the index test threshold for the primary analysis.

BP, blood pressure; NPV, negative predictive value; PPV, positive predictive value.

**Table 6:** Diagnostic performance of in-hospital daytime diastolic blood pressure as the index test for out-of-hospital daytime diastolic hypertension

In-hospital diastolic BP threshold	Sensitivity (95% CI)	Specificity (95% CI)	PPV (95% CI)	NPV (95% CI)
≥70 mm Hg	0.94 (0.86 to 0.98) 64/68	0.24 (0.17 to 0.32) 33/138	0.38 (0.31 to 0.46) 64/169	0.89 (0.75 to 0.97) 33/37
≥75 mm Hg	0.82 (0.71 to 0.91) 56/68	0.50 (0.41 to 0.59) 69/138	0.45 (0.36 to 0.54) 56/125	0.85 (0.76 to 0.92) 69/81
≥80 mm Hg	0.56 (0.43 to 0.68) 38/68	0.70 (0.62 to 0.78) 97/138	0.48 (0.37 to 0.60) 38/79	0.76 (0.68 to 0.83) 97/127
≥85 mm Hg*	0.34 (0.23 to 0.46) 23/68	0.86 (0.79 to 0.91) 118/138	0.53 (0.38 to 0.69) 23/43	0.72 (0.65 to 0.79) 118/163
≥90 mm Hg ≤110 mm Hg	0.16 (0.08 to 0.27) 11/68	0.95 (0.9 to 0.98) 131/138	0.61 (0.36 to 0.83) 11/18	0.70 (0.63 to 0.76) 131/188

* Denotes the index test threshold for the primary analysis.

BP, blood pressure; NPV, negative predictive value; PPV, positive predictive value.

### Adverse events

There were no adverse events identified from the performing of the index test or reference standard, during the study procedure.

## Discussion

### Statement of principal findings

Our study procedures identified patients at risk of community hypertension automatically from electronic hospital records and provided digitally-enabled home diagnosis. We found undiagnosed daytime community hypertension (daytime ambulatory BP ≥135 mm Hg systolic or ≥85 mm Hg diastolic) in 44% of all included patients, and 55% of patients who had ‘raised mean in-hospital daytime blood pressure’ as defined by our index test (mean daytime BP ≥135 mm Hg systolic/85 mm Hg diastolic). A further 21% had isolated night-time hypertension according to European diagnostic criteria^15^ (23% of those with ‘raised mean in-hospital daytime blood pressure’). The PPV of ‘raised mean in-hospital daytime blood pressure’ for daytime community hypertension was 58% (95% CI 45% to 65%, with sensitivity 65% (95% CI 54% to 75%), specificity 58% (95% CI 49% to 67%), and NPV 68% (95% CI 68% to 77%).

We observed that as both the systolic and diastolic BP thresholds increase, the sensitivity and NPV decrease, while specificity and PPV increase, as expected. There is no obviously dominant diagnostic cut-point for either systolic or diastolic BP where the specificity and sensitivity are both close to 100%, or indeed equal to one another.

### Study strengths and weaknesses

These findings indicate that while elevated in-hospital BP on its own has limited utility, rather than be used as a stand-alone diagnostic test, it could serve as a trigger for further BP assessment in the community after discharge. The in-hospital BP test demonstrated moderate accuracy, with a sensitivity of 65% and specificity of 58% for detecting community daytime hypertension. Thus, if this was applied as a stand-alone diagnostic test, a proportion of true cases would be undetected (limited sensitivity), and further patients without hypertension would be incorrectly classified as being hypertensive (limited specificity). This is also reflected in the PPV (55%), and the NPV (68%). Given the high rate of undiagnosed community hypertension among those without a raised in-hospital BP, a lower screening BP threshold with greater sensitivity should be considered. We observed that reducing the threshold by 5 mm Hg improved the sensitivity of the diagnostic test from 0.72 to 0.80 for systolic hypertension and 0.34 to 0.56 for diastolic hypertension. However, this was coupled with a fall in PPV from 0.38 to 0.33 for systolic hypertension and from 0.53 to 0.48 for diastolic hypertension.

Statistical approaches, for example the Youden index, have limited utility in determining the most appropriate threshold as equal weight would be given to sensitivity and specificity. A threshold would need to be considered in the context of acceptability of the test among the public, and the competing costs of increased resource use and the downstream health and cost consequences that would likely vary as the threshold for the index diagnostic test was varied.

Focusing on those patients with isolated undiagnosed night-time hypertension, we were able to identify 56% (25 of 45) of these patients as being at risk of hypertension using their day-time in-hospital BP measurements. It is likely that only the sickest patients in hospital will have multiple night-time BP readings that could be averaged to screen for nocturnal BP, and it is feasible that the index test of raised in-hospital daytime BP could also serve as a trigger for further assessment of nocturnal BP in the community after hospital discharge.

This study has a number of strengths in terms of the diagnostic accuracy study methodology. First, index test results were interpreted without knowledge of the reference standard test results, and all participants were invited to receive a reference standard test regardless of their index test result. The application of the index test, its conduct and the population it was tested in are as close as possible to how the test would be applied in practice with the target population. We can be confident that the reference standard test is likely to correctly identify the target condition, as we used the long-established and gold-standard method of hypertension diagnosis in the form of ABPM over a 24-hour period.

Risk of bias was minimised by all participants receiving the same reference standard test. The interval between the index test and reference standard (median 56 days) was sufficient that patients should have recovered from their recent hospital admission but not so prolonged that they were likely to develop new, incident hypertension during the follow-up period.

There is a notable difference between the number of participants recruited and the number who went on to receive reference testing, which is a limitation of this study; the cause for this was multifactorial. First, recruitment and follow-up of participants was suspended during 2020 at the onset of the COVID-19 pandemic to protect NHS patients and research staff. For a number of participants, the COVID-19 suspension period went beyond the period of follow-up permitted by ethical approvals and therefore could not receive reference APBM diagnostic testing. Additionally, on re-opening the study, a number of participants withdrew their participation. It is possible some participants were concerned about contracting COVID-19 through contact with healthcare professionals and medical equipment. Demographic data among those who received reference diagnostic testing and those who did not are closely aligned, and we believe that this is therefore unlikely to have led to bias in our results.

We had planned to extend the analysis to include patients who did not complete ABPM by imputing ABPM results for these individuals, but determined that this would not be rigorous given we had a greater than anticipated number of patients who did not complete ABPM.

### Strengths and weaknesses in relation to other studies

Previous work has indicated that screening for hypertension in the emergency department can identify people with previously unrecognised hypertension.^13^ This study extends that finding to a wider population admitted to hospital and reports an efficient way of identifying these individuals. A recent large observational study investigated physician management of elevated in-hospital BP.^26^ This revealed that physician management of elevated BP in hospital is not standardised, and that patients in whom new antihypertensive agents were initiated during a hospital stay had poorer clinical outcomes and more adverse events than patients who didn’t receive new antihypertensive medication during a hospital admission.^26^ The adverse outcomes observed may be due to the fact that treating all hypertensives in hospital will include marked numbers of people who will be normotensive in the community, and pharmacological management would therefore put them at unnecessary risk. It may be that antihypertensive treatment is more safely and appropriately initiated in primary care, after further BP evaluation, rather than in secondary care. This present study offers an alternative pathway to treating incident hypertension acutely in hospital and demonstrates that while a large proportion of patients will be found to be hypertensive on postdischarge BP checks, not all will be.

### Meaning of the study: possible implications for clinicians and policy makers

Our study demonstrates a novel method for digitally-enabled facility-based hypertension screening using routinely collected BP data from hospital electronic records. We have also presented a pathway for follow-up of suitable patients in primary care, delivered either digitally or more traditionally through face-to-face appointments.

Our automated study-screening algorithm analysed electronic patient records in three hospital trust settings in the UK, demonstrating applicability to a range of hospitals and their information technology systems.

While in-hospital BP measurement on its own is an imperfect predictor and should not be used as a stand-alone diagnostic test, raised measurements could serve as a trigger for further assessment of BP in the community after discharge.

### Unanswered questions and future research

Our study shows an automated system by which patients in hospital with undiagnosed hypertension may be identified, with digitally-enabled home diagnosis. Work is needed to externally validate this work prior to operating this within routine practice. Given the low numbers of participants from non-white ethnic groups, the predictive performance of the index thresholds should be tested among cohorts with greater ethnic diversity to examine their performance in a broader population.

### Study registration

The study is registered online with ISRCTN (ID ISRCTN80586284) and the protocol has previously been published elsewhere.^19^

## Supplementary material

10.1136/bmjopen-2025-107038 online supplemental file 1

10.1136/bmjopen-2025-107038 online supplemental file 2

## Data Availability

Data are available upon reasonable request.
