# Supplementary material for: Screening for hypertension in the inpatient environment (SHINE): a prospective diagnostic accuracy study among adult hospital patients
Source: BMJ Open. 2026 Jan 19;16(1):e107038. doi: 10.1136/bmjopen-2025-107038 (PMC12820866; doi:10.1136/bmjopen-2025-107038)
Supplement: online supplemental file 1 [file bmjopen-16-1-s001.docx]

**SUPPLEMENTARY RESULTS**

**Study title: Screening for Hypertension in the INpatient Environment (SHINE): A prospective diagnostic accuracy study among adult hospital patients**

**Authors:**

Laura C Armitage^a^ 0000-0002-5009-4899

Cristian Roman^b^ 0000-0002-9164-8659

Beth K Lawson^a^ 0009-0003-9524-9502

Adam Mahdi^b,c^

Christopher Biggs^d^ 0000-0003-0348-5480

Louise Young^d^ 0000-0001-9094-1733

Holly Edmondson^a,d^

Thomas R Fanshawe^a^

Lionel Tarassenko^b^ 0000-0002-0118-1646

Andrew J Farmer^a^ 0000-0002-6170-4402

Peter J Watkinson^d^ 0000-0003-1023-3927

^a^Nuffield Department of Primary Care Health Sciences, University of Oxford, Oxford, UK

^b^Institute of Biomedical Engineering, Department of Engineering Science, University of Oxford, Oxford, UK

^c^Oxford Internet Institute, University of Oxford, UK

^d^Nuffield Department of Clinical Neurosciences, University of Oxford, Oxford, UK

***Supplementary Figure 1a: Comparison of average daytime in-hospital blood pressure (systolic and diastolic) at recruitment and ambulatory daytime blood pressure in the community***


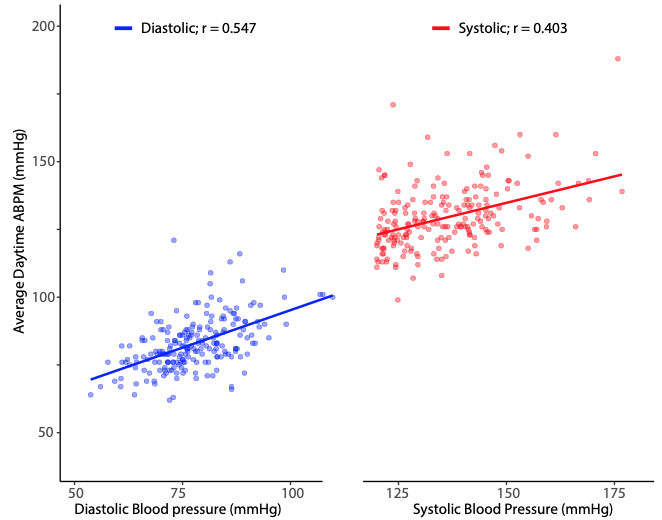


r = Pearson correlation coefficient value.

***Supplementary Figure 1b: Comparison of average daytime in-hospital blood pressure (systolic and diastolic) at recruitment and ambulatory night-time blood pressure in the community***

*
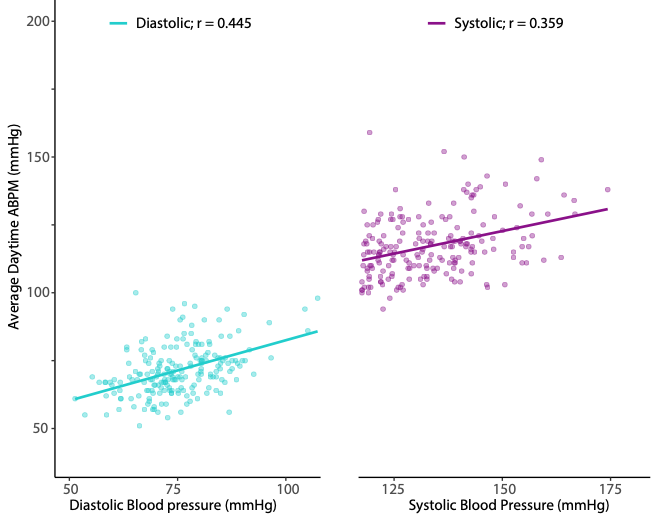
*

r = Pearson correlation coefficient value.

***Supplementary Figure 2. Receiver operator characteristic plot for in-hospital systolic blood pressure as the predictor for systolic hypertension upon reference testing with ambulatory blood pressure monitoring***


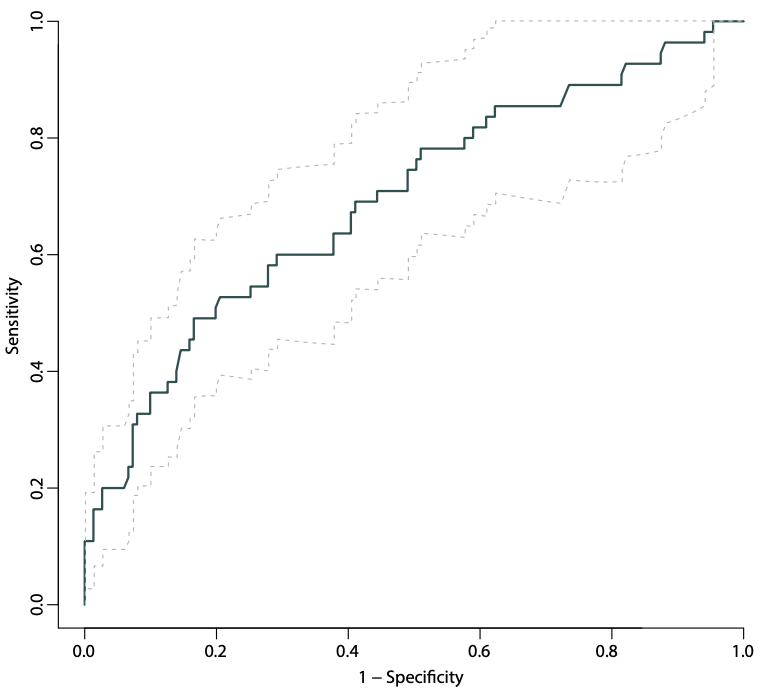


***Supplementary Figure 3. Receiver operator characteristic plot for in-hospital diastolic blood pressure as the predictor for diastolic hypertension upon reference testing with ambulatory blood pressure monitoring.***


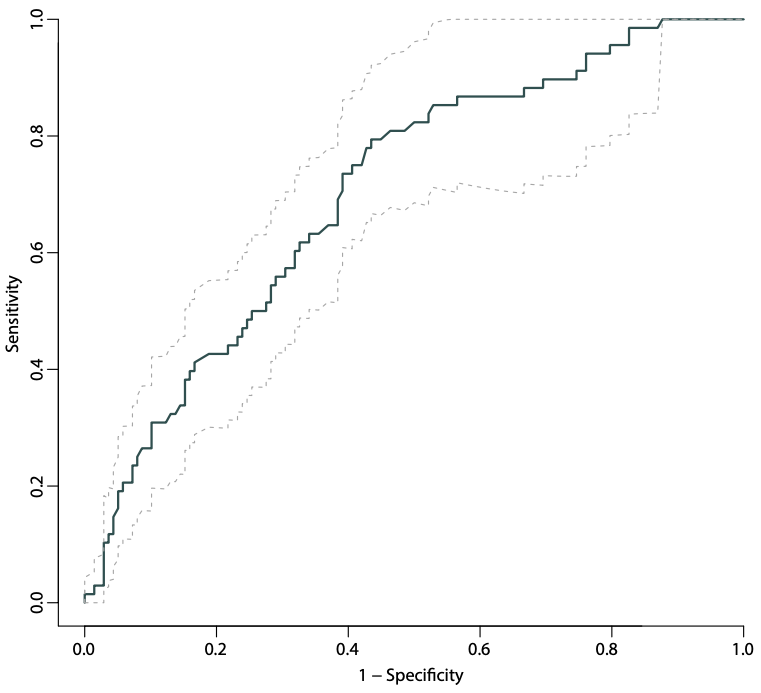


| Mean in-hospital systolic blood pressure at point of identifying eligibility | Screened (n = 17,854) | | Enrolled (n = 351) | | Completed reference-test follow up (n = 206) | |
| --- | --- | --- | --- | --- | --- | --- |
|  | Number | % | Number | % | Number | % |
| 120-129 | 7784 | 44 | 130 | 37 | 80 | 39 |
| 130-139 | 4820 | 27 | 83 | 24 | 47 | 23 |
| 140-149 | 2756 | 15 | 81 | 23 | 51 | 25 |
| 150-159 | 1507 | 8.4 | 34 | 9.7 | 17 | 8.3 |
| 160-179 | 981 | 5.5 | 22 | 6.3 | 11 | 5.3 |

***Supplementary Table S1. Proportion of patients and participants whose mean in-hospital day-time blood pressure fell into one of five pre-identified bands by screened, enrolled and completed reference testing cohorts***
