# Supplementary material for: Screening for hypertension in the inpatient environment (SHINE): a prospective diagnostic accuracy study among adult hospital patients
Source: BMJ Open. 2026 Jan 19;16(1):e107038. doi: 10.1136/bmjopen-2025-107038 (PMC12820866; doi:10.1136/bmjopen-2025-107038)
Supplement: online supplemental file 2 [file bmjopen-16-1-s002.pdf]

```
library(readxl)
library(h2o)
library(tidyr)
library(ggplot2)
library(ROCit)
library(cutpointr)
library(pROC)
library(psych)
library(dplyr)
library(predtools)
library(epiR)

Regression_df_beforeexcludingfails <- merged_dataset[c("study_id",
"Inhospital_Systolic", "Inhospital_Diastolic", "Systolic_hypertension",
"Diastolic_hypertension", "S_D_hypertension", "bl1_sex", "bl1_height", "bl1_weight",
"bl1_age", "bl2_ethnicity", "bl1_hist_db", "BMI", "abpm_fail_medical___1",
"abpm_fail_medical___2", "abpmrv_abpmreviewfail", "tf_dt_discharge",
"abpm_dttest")]
Regression_df_beforeexcludingfails %>%
  filter(!abpm_fail_medical___1 == "1") %>%
  filter(is.na(abpmrv_abpmreviewfail)) -> Regression_df

#Code sex as male / female
Regression_df$bl1_sex <- ifelse(Regression_df$bl1_sex == "1","male",
                          ifelse(Regression_df$bl1_sex == "2", "female", "NA"))

#Make categorical variables factors
Regression_df$bl1_sex <-factor(Regression_df$bl1_sex, levels = c("female",
"male"))
Regression_df$bl2_ethnicity <-factor(Regression_df$bl2_ethnicity)
Regression_df$bl1_hist_db <-factor(Regression_df$bl1_hist_db)

#Table 1 - Baseline characteristics
#Lost to follow-up patients (n=145)
describe(Regression_df_beforeexcludingfails)
table(Regression_df_lost_to_fup$bl1_sex)
table(Regression_df_lost_to_fup$bl1_hist_db)
summary(Regression_df_lost_to_fup$Inhospital_Systolic)
summary(Regression_df_lost_to_fup$Inhospital_Diastolic)

#Included patients (n=206)
describe(Regression_df)
table(Regression_df$bl1_sex)
table(Regression_df$bl1_hist_db)
summary(Regression_df$Inhospital_Systolic)
summary(Regression_df$Inhospital_Diastolic)

#Table 2 - Prevalence of Hypertension
#Calculate patients who are hypertensive
```

```
merged_dataset %>%
  filter(!abpm_fail_medical___1 == "1") %>%
  filter(is.na(abpmrv_abpmreviewfail)) %>%
  filter(is.na(Systolic_hypertension) == "FALSE") %>%
  filter(is.na(Inhospital_Systolic) == "FALSE")-> merged_dataset2

merged_dataset2$Systolic_hypertension <-
factor(merged_dataset2$Systolic_hypertension)
merged_dataset2$Diastolic_hypertension <-
factor(merged_dataset2$Diastolic_hypertension)

merged_dataset2$Raised_BP_inhospital <-
ifelse(merged_dataset2$Inhospital_Systolic >=135 |
merged_dataset2$Inhospital_Diastolic >=85, "1", "0")
merged_dataset2$Daytime_HTN <- ifelse(merged_dataset2$abpm_av_sys_day
>=135 | merged_dataset2$abpm_av_dia_day >=85, "1", "0")
merged_dataset2$Nighttime_HTN <- ifelse(merged_dataset2$abpm_av_sys_night
>=120 | merged_dataset2$abpm_av_dia_night >=70, "1", "0")
merged_dataset2$Day_or_night_HTN <- ifelse(merged_dataset2$Daytime_HTN ==
"1" | merged_dataset2$Nighttime_HTN == "1", "1", "0")

merged_dataset2$Raised_BP_inhospital <-
factor(merged_dataset2$Raised_BP_inhospital, levels = c("1", "0"))
merged_dataset2$Day_or_night_HTN <-
factor(merged_dataset2$Day_or_night_HTN, levels = c("1", "0"))

tab <- table(merged_dataset2$Raised_BP_inhospital,
merged_dataset2$Day_or_night_HTN, dnn = c("Above threshold", "Outcome"))
(epi.tests(tab, method = "exact", digits = 2, conf.level = 0.95))

ifelse(merged_dataset2$abpm_av_sys_day >= 160 |
merged_dataset2$abpm_av_dia_day >= 105, "Severe",
       ifelse((merged_dataset2$abpm_av_sys_day >= 150 &
merged_dataset2$abpm_av_sys_day < 160) | (merged_dataset2$abpm_av_dia_day
>= 95 & merged_dataset2$abpm_av_dia_day < 105), "Stage 2",
             ifelse((merged_dataset2$abpm_av_sys_day >= 135 &
merged_dataset2$abpm_av_sys_day < 150) | (merged_dataset2$abpm_av_dia_day
>= 85 & merged_dataset2$abpm_av_dia_day < 95), "Stage 1",
                   ifelse(merged_dataset2$abpm_av_sys_day <135 |
merged_dataset2$abpm_av_dia_day <85, "Normotensive", "NA")))) ->
merged_dataset2$Daytime_HTN_stages
table(merged_dataset2$Daytime_HTN_stages)

#Table 3 - 2x2 table
tab <- table(merged_dataset2$Raised_BP_inhospital,
Regression_df3$S_or_D_HTN, dnn = c("Above threshold", "Outcome"))
(epi.tests(tab, method = "exact", digits = 2, conf.level = 0.95))

#Table 4 - 2x2 table
tab <- table(merged_dataset2$Raised_BP_inhospital,
```

```
merged_dataset2$Day_or_night_HTN, dnn = c("Above threshold", "Outcome"))
(epi.tests(tab, method = "exact", digits = 2, conf.level = 0.95))

#Supplementary Figure 1a - comparison of average daytime inhospital blood
pressure at recruitment and ambulatory daytime blood pressure in the community
Systolic_df <- data.frame(Inhospital = merged_dataset_filt$Inhospital_Systolic,
                          ABPM = merged_dataset_filt$best_day_SBP)
Systolic_df["Systolic_diastolic"] = "Systolic"
as.numeric(Systolic_df$ABPM) -> Systolic_df$ABPM
cor(Systolic_df$Inhospital, Systolic_df$ABPM, method = "pearson")
Diastolic_df <- data.frame(Inhospital = merged_dataset_filt$Inhospital_Diastolic,
                           ABPM = merged_dataset_filt$best_day_DBP)
Diastolic_df["Systolic_diastolic"] = "Diastolic"
as.numeric(Diastolic_df$ABPM) -> Diastolic_df$ABPM
cor(Diastolic_df$Inhospital, Diastolic_df$ABPM, method = "pearson")
rbind(Systolic_df, Diastolic_df) -> df1
df1 %>%
  ggplot(aes(x=Inhospital, y=ABPM, color = Systolic_diastolic)) +
  geom_point(alpha=0.4)+
  scale_color_manual(values = c("Blue", "Red")) +
  geom_smooth(method = "lm", se = F) +
  ylim(40,200) +
  xlim(40,200) +
  theme_classic()

#Supplementary figure 1b - comparison of average daytime inhospital blood
pressure at recruitment and ambulatory night time blood pressure in the community
merged_dataset %>%
  filter(!is.na(abpm_av_sys_night)) %>%
  filter(abpm_av_sys_night >20) -> merged_dataset_filt

Systolic_df <- data.frame(Inhospital = merged_dataset_filt$Inhospital_Systolic,
                          ABPM = merged_dataset_filt$abpm_av_sys_night)
Systolic_df["Systolic_diastolic"] = "Systolic"
cor(Systolic_df$Inhospital, Systolic_df$ABPM, method = "pearson")
Diastolic_df <- data.frame(Inhospital = merged_dataset_filt$Inhospital_Diastolic,
                           ABPM = merged_dataset_filt$abpm_av_dia_night)
Diastolic_df["Systolic_diastolic"] = "Diastolic"
cor(Diastolic_df$Inhospital, Diastolic_df$ABPM, method = "pearson")
rbind(Systolic_df, Diastolic_df) -> df1
df1 %>%
  ggplot(aes(x=Inhospital, y=ABPM, color = Systolic_diastolic)) +
  geom_point(alpha=0.4)+
  scale_color_manual(values = c("cyan3", "magenta4")) +
  geom_smooth(method = "lm", se = F) +
  ylim(40,200) +
  xlim(40,200) +
  theme_classic()

#Supplementary figure 2 - receiver operating characteristic curve of inhospital
```

systolic BP for prediction of systolic hypertension in the community

```
model <- glm(Regression_df3$Systolic_hypertension ~
Regression_df3$Inhospital_Systolic, family = "binomial", na.action=na.exclude)
Regression_df3$model <- predict(model, type = 'response')
score <- (Regression_df3$model)
class <- Regression_df3$Systolic_hypertension
SBP_only <- rocit(score = score, class = class)
ci.rocSBPonly <- ciROC(SBP_only, level = 0.95)
plot(ci.rocSBPonly)
```

#Supplementary figure 3 - receiver operating characteristic curve of inhospital diastolic BP for prediction of diastolic hypertension in the community

```
model <- glm(Regression_df3$Diastolic_hypertension ~
Regression_df3$Inhospital_Diastolic, family = "binomial", na.action=na.exclude)
Regression_df3$model <- predict(model, type = 'response')
score <- (Regression_df3$model)
class <- Regression_df3$Diastolic_hypertension
DBP_only <- rocit(score = score, class = class)
ci.rocDBPonly <- ciROC(DBP_only, level = 0.95)
plot(ci.rocDBPonly)
```

#Supplementary table S1 - proportion of patients and participants whose mean inhospital day time blood pressure fell into pre-identified bands

```
table(merged_dataset$Inclusion, merged_dataset$bl1_band)
```

#Tables 5 and 6 - Diagnostic performance of inhospital day time BP as the index test for out of hospital daytime systolic or diastolic HTN

```
CI_table_function <- function(threshold_data, outcome_data, threshold_value){
  Above_threshold <- ifelse(threshold_data >= threshold_value, "1", "0")
  df <- data.frame(Above_threshold, outcome_data)
  df$Above_threshold <- factor(df$Above_threshold, levels = c(1,0))
  df$outcome_data <- factor(df$outcome_data, levels = c(1,0))
  tab <- table(df$Above_threshold,df$outcome, dnn = c("Above threshold",
"Outcome"))
  return(epi.tests(tab, method = "exact", digits = 2, conf.level = 0.95))
}
```

#Apply this to each of the blood pressure thresholds for systolic (120, 125, 130, 135, 140, 150, 160) and diastolic (70, 75, 80, 85, 90) hypertension to derive sensitivity, specificity, positive predictive value (PPV) and negative predictive value (NPV), examples for systolic and diastolic below.

```
CI_table_function(threshold_data = Regression_df2$Inhospital_Systolic,
              threshold_value = 120,
              outcome_data = Regression_df2$S_HTN) #Run for series of systolic BP
thresholds

CI_table_function(threshold_data = Regression_df2$Inhospital_Diastolic,
              threshold_value = 70,
              outcome_data = Regression_df2$D_HTN) #Run for series of diastolic BP
```

thresholds
