# Supplementary material for: Screening for hypertension in the inpatient environment (SHINE): a prospective diagnostic accuracy study among adult hospital patients
Source: BMJ Open. 2026 Jan 19;16(1):e107038. doi: 10.1136/bmjopen-2025-107038 (PMC12820866; doi:10.1136/bmjopen-2025-107038)
Supplement: Supplementary file 3 [file bmjopen-2025-107038.reviewer_comments.pdf]

PEER REVIEW HISTORY

BMJ Open publishes all reviews undertaken for accepted manuscripts. Reviewers are asked to complete a checklist review form and are provided with free text boxes to elaborate on their assessment. These free text comments are reproduced below.

ARTICLE DETAILS

Title (Provisional)

Screening for Hypertension in the INpatient Environment (SHINE): A prospective diagnostic accuracy study among adult hospital patients

Authors

Armitage, Laura Catherine; Roman, Cristian; Lawson, Beth K; Mahdi, Adam; Biggs, Christopher; Young, Louise; Edmundson, Holly; Fanshawe, Thomas; Tarassenko, Lionel; Farmer, Andrew; Watkinson, Peter J

VERSION 1 - REVIEW

**Reviewer**	**1**
**Name**	**de la Sierra, Alejandro**
**Affiliation**	**Univ Barcelona**
**Date**	**06-Aug-2025**
**COI**	**None**

This study evaluates the accuracy of an in-hospital elevated BP (135/85) with respect to a diagnosis based on ABPM performed about 8 weeks after the in-hospital measurement. The authors raise several conclusions, including 1st: undiagnosed hypertension is common in hospitalized patients, 2nd: hospital BP is a good tool for identifying those with more probabilities to be hypertensive, not only by having ambulatory daytime hypertension (NICE guidelines) but also isolated nocturnal hypertension (as EU guidelines consider when only nocturnal BP is elevated by means of 24-h ABPM recording).

General comment:

I agree with the authors' first conclusion, i.e. the prevalence of undiagnosed hypertension among adults suffering a hospital admission is high, but more skeptical regarding the accuracy of the index test (in-hospital BP values). Looking at values shown in table 3, sensitivity and specificity are only moderately higher than 50% (65% for sensitivity and 58% for specificity), Pearson's correlation coefficients are also moderate (supplementary figure 1 a) and AUROC are 0.69 and 0.70. In fact, the proportion of hypertensive individuals among those with in-hospital BP > 135/85 was 59/107; 55%. However, the proportion of hypertensives among those with in-hospital BP < 135/85 was 32/99; 32%, indicating that

even with normal values of in-hospital BP, there is still one third of individuals with undiagnosed hypertension. Obviously, both specificity and PPV increased if isolated nocturnal hypertension was also computed. However, it seems that the proportion of patients with this diagnosis was identical among those with or without elevated in-hospital BP. I think the authors should be less strong when raising their conclusions. In addition, this overlap should be discussed and included as a limitation.

Other aspects:

1. A secondary objective was to explore the accuracy of different in-hospital BP thresholds for the diagnosis of ambulatory hypertension. However, the results of this objective are not even mentioned in the abstract, very scarcely in the results, and only contained in 2 supplementary tables. Maybe by using ROC curves (Youden index), the authors could obtain BP threshold with a better accuracy in the prediction of a hypertension diagnosis.

2. The time between hospital discharge and the ABPM procedure is very important, especially in a group of patients who were hospitalized by different causes. The authors comment in the discussion that the median between index and reference was 59 days, enough to recover from the consequences of hospitalization. In the hypertension clinic, it is often observed that hypertensive patients who suffer a hospitalization have their BP (and antihypertensive requirements) considerably reduced during several weeks after discharge (obviously very dependent on the cause of hospitalization). In this view, the lower limit for ABPM (4 weeks) could be affected by this phenomenon. I would suggest to adjust for the impact of the time between BP measurements and evaluating if the accuracy is different depending on such time.

3. Was in-hospital BP measured while the patients was seated, supine, or irrespective of his/her position?

4. Definitions for stage 1, stage 2, and severe hypertension would better fit in the methods section.

5. In the abstract, the definition of hypertension should include grade 3 HT the day of ABPM, excluding those patients from this procedure.

Reviewer **2**

Name **Kurth, Tobias**

Affiliation **Charité Universitätsmedizin Berlin, Institute of Public Health**

Date **13-Sep-2025**

COI **None**

In this prospective cohort study, Armitage and colleagues aimed to investigate the accuracy and performance of current UK community BP thresholds for diagnosing hypertension in the hospital setting. In 206 participants who underwent ambulatory blood pressure monitoring as the reference standard, in-hospital daytime blood pressure showed a sensitivity of 65% and specificity of 58% for detecting community daytime hypertension, with a positive predictive value of 55% and a negative predictive value of 68%. The authors concluded that undiagnosed hypertension is frequent among hospitalized patients, especially those with elevated in-hospital blood pressure, and post-discharge community blood pressure measurement could help detect previously unrecognized cases.

While I applaud the authors for conducting this study, and the results may inform health care providers about blood pressure management, several aspects limit the interpretability.

1. Of the 5823 patients deemed eligible, only 351 could be included, of which another 145 patients did not participate for various reasons. Estimates of prevalence or measures of predictive value or accuracy are therefore only very limitedly interpretable. I understand that there is nothing that the authors can do to change this.

2. The in-hospital blood pressure test showed only moderate sensitivity (65%) and specificity (58%) for detecting community daytime hypertension. This means that a substantial proportion of true cases were missed (limited sensitivity), and many patients without hypertension were incorrectly classified as positive (limited specificity). The positive predictive value was modest (55%), indicating that nearly half of those flagged as hypertensive in the hospital did not have hypertension when assessed with ambulatory blood pressure monitoring (ABPM). The negative predictive value was slightly higher (68%), suggesting that a normal in-hospital blood pressure made community hypertension somewhat less likely but did not reliably exclude it. These findings indicate that elevated in-hospital blood pressure alone is an imperfect predictor and should not be used as a stand-alone diagnostic test, but could serve as a trigger for further assessment using ABPM after discharge. When reading the discussion and interpretation of the study, these aspects are not adequately addressed.

3. I may have missed it, but the authors do not report the c-statistic of their ROC curves. From the figures and other results, I do not expect them to be high.

4. The authors should provide the RStudio versions that were used for the analyses.

5. The details provided in the sample size sections are not sufficient to reproduce this calculation.

VERSION 1 - AUTHOR RESPONSE

Reviewer: 1
Dr. Alejandro de la Sierra, Univ Barcelona

Comments to the Author:
This study evaluates the accuracy of an in-hospital elevated BP (135/85) with respect to a diagnosis based on ABPM performed about 8 weeks after the in-hospital measurement. The authors raise several conclusions, including 1st: undiagnosed hypertension is common in hospitalized patients, 2nd: hospital BP is a good tool for identifying those with more probabilities to be hypertensive, not only by having ambulatory daytime hypertension (NICE guidelines) but also isolated nocturnal hypertension (as EU guidelines consider when only nocturnal BP is elevated by means of 24-h ABPM recording).

General comment:
I agree with the authors' first conclusion, i.e. the prevalence of undiagnosed hypertension among adults suffering a hospital admission is high, but more skeptical regarding the accuracy of the index test (in-hospital BP values). Looking at values shown in table 3, sensitivity and specificity are only moderately higher than 50% (65% for sensitivity and 58% for specificity), Pearson's correlation coefficients are also moderate (supplementary figure 1 a) and AUROC are 0.69 and 0.70. In fact, the proportion of hypertensive individuals among those with in-hospital BP > 135/85 was 59/107; 55%. However, the proportion of hypertensives among those with in-hospital BP < 135/85 was 32/99; 32%, indicating that even with normal values of in-hospital BP, there is still one third of individuals with undiagnosed hypertension. Obviously, both specificity and PPV increased if isolated nocturnal hypertension was also computed. However, it seems that the proportion of patients with this diagnosis was identical among those with or without elevated in-hospital BP. I think the authors should be less strong when raising their conclusions. In addition, this overlap should be discussed and included as a limitation.

Author's response:
Thank you, we have revised the conclusion to be less strong, and focussed more on the limited sensitivity and specificity in the 'Study Strengths and weaknesses' section of the discussion,.

Other aspects:
1. A secondary objective was to explore the accuracy of different in-hospital BP thresholds for the diagnosis of ambulatory hypertension. However, the results of this objective are not even mentioned in the abstract, very scarcely in the results, and only contained in 2 supplementary tables. Maybe by using ROC curves (Youden index), the authors could obtain BP threshold with a better accuracy in the prediction of a hypertension diagnosis.

Author's response:
Thank you for this suggestion. We have made some changes to address these points. We have endeavoured to keep within the word count for the abstract and thus have only included the findings of the primary outcome within this, whilst adding small changes to the methods and conclusions that have been requested for this revision. We have moved supplementary tables S2a and S2b which present the sensitivity, specificity, PPV and NPV for a range of

exploratory thresholds into the main manuscript (now tables 5 and 6). We have left the ROC curves in the supplementary files, as we believe the AUROC value for both of the ROC curves is the most important finding of interest for the reader, but that readers should be able to refer to the visual presentation of the ROC if they wish. We carefully considered using the Youden index, but feel its clinical usefulness is limited, as it is a purely statistical method of identifying a threshold that gives equal weighting to sensitivity and specificity, rather than one that considers the competing clinical risks between false negatives that arise with increasing specificity and false positives that arise with increasing sensitivity. We have added a comment regarding this to the discussion under the 'Strengths and weaknesses' section.

2. The time between hospital discharge and the ABPM procedure is very important, especially in a group of patients who were hospitalized by different causes. The authors comment in the discussion that the median between index and reference was 59 days, enough to recover from the consequences of hospitalization. In the hypertension clinic, it is often observed that hypertensive patients who suffer a hospitalization have their BP (and antihypertensive requirements) considerably reduced during several weeks after discharge (obviously very dependent on the cause of hospitalization). In this view, the lower limit for ABPM (4 weeks) could be affected by this phenomenon. I would suggest to adjust for the impact of the time between BP measurements and evaluating if the accuracy is different depending on such time.

Author's response:
Thank you for this interesting suggestion. We have now compared the time to follow up for those correctly classified (true positives plus true negatives) vs. those incorrectly classified (false positives and false positives). Interestingly we found that the time to follow up ABPM was shortest in those correctly classified. These additional results are included at the top of page 13 of the manuscript.

3. Was in-hospital BP measured while the patients was seated, supine, or irrespective of his/her position?

Author's response:
Irrespective of position – we did not control any element of the in-hospital BP; it was measured as part of routine care. We have added more detail regarding this under the 'Diagnostic test methods – Index Diagnostic Test' subheading in the main manuscript. We believe this makes the applicability as close as possible to a real-life setting rather than a controlled study setting

4. Definitions for stage 1, stage 2, and severe hypertension would better fit in the methods section.

Author's response:
Thank you, we agree and have moved this to the methods section.

5. In the abstract, the definition of hypertension should include grade 3 HT the day of ABPM, excluding those patients from this procedure.

Author's response:
Thank you, we have now added this to the abstract.

Reviewer: 2
Dr. Tobias Kurth, Charité Universitätsmedizin Berlin
Comments to the Author:
In this prospective cohort study, Armitage and colleagues aimed to investigate the accuracy and performance of current UK community BP thresholds for diagnosing hypertension in the hospital setting. In 206 participants who underwent ambulatory blood pressure monitoring as the reference standard, in-hospital daytime blood pressure showed a sensitivity of 65% and specificity of 58% for detecting community daytime hypertension, with a positive predictive value of 55% and a negative predictive value of 68%. The authors concluded that undiagnosed hypertension is frequent among hospitalized patients, especially those with elevated in-hospital blood pressure, and post-discharge community blood pressure measurement could help detect previously unrecognized cases.

While I applaud the authors for conducting this study, and the results may inform health care providers about blood pressure management, several aspects limit the interpretability.

1. Of the 5823 patients deemed eligible, only 351 could be included, of which another 145 patients did not participate for various reasons. Estimates of prevalence or measures of predictive value or accuracy are therefore only very limitedly interpretable. I understand that there is nothing that the authors can do to change this.

Authors' response:
We agree this is a limitation of the study and have acknowledged the limited number of participants who completed the protocol in the paragraph at the bottom of page 19/top of page 20.

2. The in-hospital blood pressure test showed only moderate sensitivity (65%) and specificity (58%) for detecting community daytime hypertension. This means that a substantial proportion of true cases were missed (limited sensitivity), and many patients without hypertension were incorrectly classified as positive (limited specificity). The positive predictive value was modest (55%), indicating that nearly half of those flagged as hypertensive in the hospital did not have hypertension when assessed with ambulatory blood pressure monitoring (ABPM). The negative predictive value was slightly higher (68%), suggesting that a normal in-hospital blood pressure made community hypertension somewhat less likely but did not reliably exclude it. These findings indicate that elevated in-hospital

blood pressure alone is an imperfect predictor and should not be used as a stand-alone diagnostic test, but could serve as a trigger for further assessment using ABPM after discharge. When reading the discussion and interpretation of the study, these aspects are not adequately addressed.

Authors' response
Thank you. We have discussed this more fully now in the manuscript and also included your very salient point about this being useful as a trigger but not a stand-alone diagnostic test in the conclusions of both the main manuscript and the abstract, as well as the 'Meaning of the study: possible implications for clinicians and policy makers' subheading.

3. I may have missed it, but the authors do not report the c-statistic of their ROC curves. From the figures and other results, I do not expect them to be high.

Authors' response
Thank you. We have already included this but used the terms 'Area Under the Receiver Operating Characteristic Curve' and 'AUROC' for short rather than 'C-statistic'. This was 0.69 (95% CI 0.61 to 0.78) for in-hospital systolic BP as the predictor for community systolic hypertension and 0.70 (95% CI 0/62 to 0.78) for in-hospital diastolic BP as the predictor for community diastolic hypertension.

4. The authors should provide the RStudio versions that were used for the analyses.

Authors' response
This is already reported under the 'Analysis' subheading in the methods. (v4.1.2; R Core Team 2021).

5. The details provided in the sample size sections are not sufficient to reproduce this calculation.

Authors' response
Apologies, the 5.36% margin error was a typo and should have read 5.52%. This has now been amended in the manuscript and others should be able to preproduce this calculation.

VERSION 2 - REVIEW

**Reviewer**	**1**
**Name**	**de la Sierra, Alejandro**
**Affiliation**	**Univ Barcelona**
**Date**	**17-Nov-2025**

COI

The authors have satisfactorily answered all my comment. The manuscript is improved and I do not have further comments
